# Overexpressed miR-200a promotes bladder cancer invasion through direct regulating Dicer/miR-16/JNK2/MMP-2 axis

**DOI:** 10.1038/s41388-019-1120-z

**Published:** 2019-11-26

**Authors:** Rui Yang, Jiheng Xu, Xiaohui Hua, Zhongxian Tian, Qipeng Xie, Jingxia Li, Guosong Jiang, Mitchell Cohen, Hong Sun, Chuanshu Huang

**Affiliations:** Department of Environmental Medicine, New York University School of Medicine, 341 East 25th Street, New York, NY 10010 USA

**Keywords:** Bladder cancer, Extracellular matrix, Bladder cancer, Extracellular matrix

## Abstract

Invasive bladder cancer (BC) is one of the most lethal malignant urological tumors. Although miR-200a has been reported as an onco-miRNA that targets the *PTEN* gene in endometrioid carcinoma, its biological significance in BC invasion has been poorly explored. In the current study, we found that miR-200a was markedly overexpressed in both human BC tissues and BBN-induced muscle-invasive BC tissues. We further showed that miR-200a overexpression specifically promoted human BC cell invasion, but not migration, *via* transcriptional upregulation of matrix metalloproteinase (MMP)-2. Mechanistic studies indicated that the increased phosphorylation of c-Jun mediated the increasing levels of *MMP-2* mRNA transcription. Further investigation revealed that Dicer was decreased in miR-200a overexpressed BC cells; this resulted in inhibition of miR-16 maturation and consequently led to increased JNK2 protein translation and c-Jun activation. Taken together, the studies here showed that miR-200a overexpression inhibited Dicer expression, in turn, resulted in inhibition of miR-16 maturation, leading to upregulation of JNK2 expression, c-Jun phosphorylation, *MMP-2* transcription and, ultimately, BC invasion. Collectively, these results demonstrate that miR-200a is an onco-miRNA that is a positive regulator for BC invasion. This finding could be very useful in the ongoing development of new strategies to treat invasive BC patients.

## Introduction

Bladder cancer (BC) is one of the common malignant urological tumors [1–4]. The most common type of BC is non-muscle-invasive bladder cancer (NMIBC), which accounts for 75% of newly diagnosed BC, while muscle-invasive bladder cancer (MIBC) accounts for 25% of all new diagnoses [5]. Despite the fact that NMIBC can be managed by a combination of transurethral resection and intravesical chemotherapy, >50–70% of NMIBC will recur. Eventually, 10–20% of recurrent tumors will invade local muscles or metastasize [6]. The multimodal therapy, the combination of surgery and radio-chemotherapy, is a standard treatment for MIBC [7], but the 5-year survival rate is very poor [8]. In addition, the refractory bladder tumor is highly related to its ability to become invasive [5]. Thus, discovering and understanding the underlying mechanisms of BC invasion would be a key step in the development of novel therapeutic strategies to prevent or manage BC.

microRNA(miRNA) is small noncoding RNA that plays important roles in various human cancers, including BC. Control of their expression is important in cells, i.e., aberrant expression of miRNA may result in them acting as either tumor suppressors or oncogenes that lead to progressive status of cancers [9]. The miR-200 family, which includes miR-200a, miR-200b, miR-200c, miR-141, and miR-429, has been reported to be involved in several different aspects of cancer biology, including epithelial-to-mesenchymal transition (EMT), tumor angiogenesis, and tumor chemotherapy resistance; these properties appear to be due, in part, to the regulation of expression of some important target genes by miR-200 members [10–12]. Notably, overexpressed miR-200a shows an inhibition of EMT by directly targeting and downregulating ZEB1 and ZEB2 *via* miR-200a-binding sites located within their 3′UTRs in normal murine mammary epithelial cells [12, 13]; even so, its potential role in human BC is poorly understood.

There are several means by which the miRNA expression in cells can be regulated. For example, Dicer is a cytoplasmic RNase III-type endonuclease that can regulate miRNA maturation by participating in miRNA intracellular processes and transfers. Dicer expression levels has been reported to be upregulated in prostate adenocarcinoma [14], but downregulated in ovarian [15] and lung cancers [16]. Interestingly, Dicer expression levels have been correlated with poor prognoses among cancer patients [17]. Because Dicer can catalyze the biosynthesis of miRNA and siRNA, this could regulate the expression of numerous genes. Accordingly, expression of the *Dicer* gene itself may be a highly-regulated process [18, 19]. Some studies indicate that discrepancies in/dysregulation of Dicer expression among various tumor types are attributed to tissue-specific differences/to degree of aggressiveness of the given cancer [20, 21]. Dicer has been reported to be downregulated in human BCs [22], which may result in increased cell proliferation in BC T24 cells [23]. However, very little is known about the function of Dicer in BC invasion.

The importance of Dicer in BC migration and invasion in situ might be attributed to its downstream effects on proteins that appear to have an impact on these properties. Some studies have indicated that alterations in matrix metalloproteinase-2 (MMP-2) expression are often associated with overall metastatic potentials of many types of cancers, including breast [24], colorectal [24], and ovarian cancers [25]. Interestingly, earlier studies from our laboratories show that MMP-2 overexpression was crucial for human BC invasive capacity [26]. Our other studies indicate that the inhibition of the MMP-2 expression by anti-cancer agent isorhapontigenin (ISO) significantly attenuated both BC invasion in vitro and highly invasive BC formation in vivo [27]. Together, these findings suggest that MMP-2 plays a key role in BC invasion in situ. How these might be related back to Dicer expression is not clear, and thus was a focus of the study reported here.

In the present study, it was seen that miR-200a overexpression could reduce Dicer protein levels. This resulted in the inhibition of miR-16 maturation and a subsequent increase in JNK2 protein translation/expression. The latter resulted in increases in cellular levels of phosphorylated c-Jun level. As a result, there was a promotion of *MMP-2* gene transcription. In the end, all of these changes gave rise to increases in BC cell invasion. Beyond that important result, the other findings here about miR-200a acting as an onco-miRNA that promotes BC cell invasion could pave the way for its potential use as a biomarker in BC diagnosis and/or as a therapeutic target in novel treatments of MIBC patients.

## Results

### miR-200a expression was upregulated in both human and mouse invasive BC tissues, and the increased miR-200a expression promoted invasion by BC cells

**Fig. 1:**
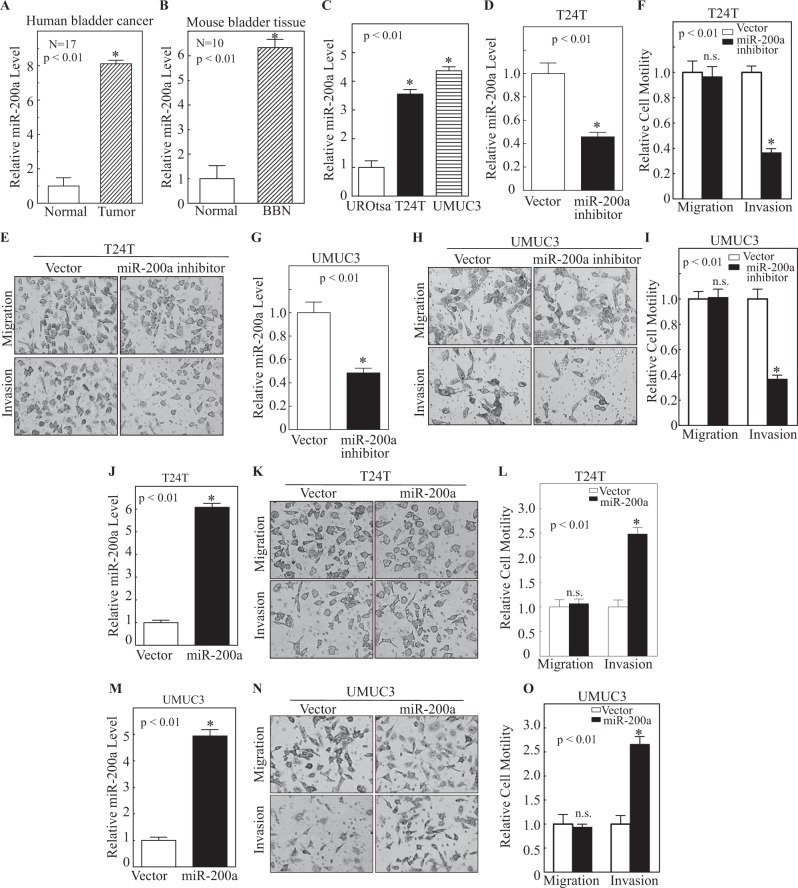
Overexpression of miR-200a in human BCs and BBN-treated mouse invasive BCs, and its specific promotion of cell invasion in human invasive BC cell lines. **a**, **b** miR-200a expression levels analyzed by real-time PCR in human and mouse BC tissues. **c** miR-200a expression was analyzed by real-time PCR in UROtsa, T24T, and UMUC3 cell lines. **d**–**i** miR-200a expression analyzed by real-time PCR in T24T(Vector) vs. T24T (miR-200a inhibitor) (**d**) and UMUC3(Vector) vs. UMUC3(miR-200a inhibitor) (**g**) cells. Invasive abilities of T24T (Vector) vs. T24T (miR-200a inhibitor) (**e**, **f**) and UMUC3(Vector) vs. UMUC3(miR-200a inhibitor) (**h**, **i**) were analyzed in Invasion Chambers. **j**–**o** miR-200a expression was analyzed by real-time PCR in T24T (Vector) vs. T24T (miR-200a) (**j**) and UMUC3(Vector) vs. UMUC3(miR-200a inhibitor) (**m**) cells. The invasive abilities of T24T(Vector) vs. T24T(miR-200a) cells and UMUC3(Vector) vs. UMUC3(miR-200a) cells were determined in invasion chambers; and the results were presented as relative invasion in the two paired transfectants, respectively (**k**, **l**, **n**, **o**). Bars represent means ± SD of three independent experiments. *p < 0.01

The members of the miR-200 family have been reported to repress the EMT and therefore suppress cancer invasion [9, 28]. To explore the potential role of miR-200 in BC invasion, we first analyzed the potential change of miR-200 family in human BCs in comparison to normal human bladder tissues in TCGA database and the results showed that the expressions of miR-200a, miR-200b, miR-429, and miR-141 were remarkably upregulated in comparison to grouped normal human bladder tissues or their paired adjacent normal bladder tissues, whereas there was no significant alteration of miR-200c between human bladder tumors and normal bladder tissues (Fig. S1). Considering workload of investigating each member of miR-200 family, current studies first focused on exploring potential contribution of miR-200a to human BC invasion. To confirm the unexpected finding of miR-200a upregulation in human BCs, we also evaluated the expression status of miR-200a in both human BC tissues and N-butyl-N-(4-hydroxybutyl) nitrosamine (BBN)-induced mouse invasive BC tissues. The results showed that the miR-200a expression level was also remarkably increased in human BC samples in comparison to their adjacent normal bladder tissues (Fig. 1a). Consistently, the miR-200a expression level in BBN-induced mouse MIBCs was also much higher than those of the vehicle-treated group (Fig. 1b). In addition, the miR-200a expression level was higher in human BC cells (T24T and UMUC3) than that in normal urothelial cells (UROtsa) (Fig. 1c). Thus, miR-200a is consistently increased in both BC tissues and cell lines.

To identify the role(s) of miR-200a in BC invasion, miR-200a was knocked down in both T24T and UMUC3 cells and analyzed for cell migration and invasion properties. The results indicated that introduction of miR-200a inhibitor specifically attenuated the miR-200a expression without affecting other members of miR-200 family (Fig. 1d & S2). The knockdown of miR-200a also specifically inhibited cell invasion without affecting cell migration in both T24T and UMUC3 cells (Fig. 1e-i), suggesting that miR-200a plays an essential role in the promotion of invasion in human BC cells. Further, the expressing construct containing miR-200a was introduced into T24T and UMUC3 cells to establish the stable transfectants. Ectopic expression of miR-200a was validated by real-time PCR in both T24T and UMUC3 cells (Fig. 1j, m). Consistent with miR-200a promotion of invasion in human BC cells observed in using knockdown approach, overexpression of miR-200a also specifically resulted in a remarkable increase of cell invasion, but not migration, in both T24T (Fig. 1k, l) and UMUC3 (Fig. 1n, o) cells. To exclude the possibility that miR-200a-regulated cell invasion was due to its effect on cell proliferation, we also determined the effect of miR-200a overexpression on T24T cell proliferation. The results indicated that the cells expressing miR-200a did not show a significant increase in cell proliferation within 36 h of cell culture, whereas it did show an increased cell proliferation at 48 h (Fig. S3). Given that cell invasion was observed at 24 h, miR-200a-regulated cell invasion was not due to its effect on cell proliferation. Thus, the results obtained from both gain- and loss-of-function studies revealed that miR-200a is a positive regulator for human BC invasion.

### MMP-2 functioned as miR-200a downstream effector for promotion of BC cell invasion

**Fig. 2:**
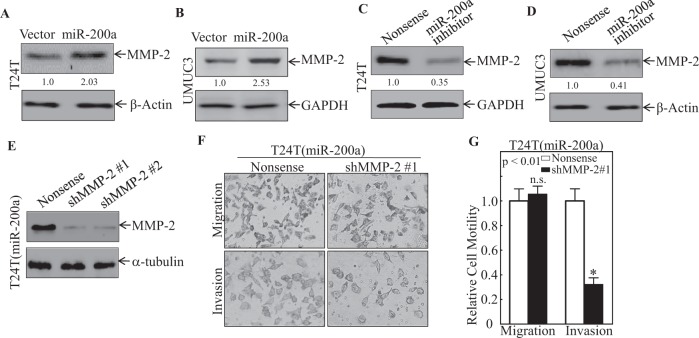
MMP-2 was miR-200a downstream effector responsible for promoting BC cell invasion. **a**, **b** MMP-2 expression was analyzed by western blot in T24T(Vector) vs. T24T(miR-200a) and UMUC3(Vector) vs. UMUC3(miR-200a) cells. Densitometric quantification of MMP-2 (relative to loading control) was presented as shown in the figures. **c**, **d** miR-200a nonsense and inhibitor were stably transfected into T24T and UMUC3 cells. MMP-2 expression was analyzed by western blot. **e** shMMP-2 and Nonsense control were stably transfected into T24T(miR-200a) cells; MMP-2 expression was analyzed by western blot. T24T(miR-200a/Nonsense) vs. T24T(miR-200a/shMMP-2) cell invasion abilities were evaluated using Invasion Chambers (**f**); relative invasion was plotted (**g**); and Bars represent means ± SD of three independent experiments. *p < 0.01

Since our previous studies indicate that MMP-2 plays a key role in human BC invasion [26, 29], the potential effect of miR-200a overexpression on MMP-2 expression was evaluated in both T24T and UMUC3. The results showed that miR-200a overexpression led to a profound upregulation of MMP-2 levels in both T24T and UMUC3 cells compared with their vector (Fig. 2a, b). Accordingly, the knockdown of the miR-200a expression by its specific shRNA caused a dramatic attenuation of the MMP-2 expression in both cell lines (Fig. 2c, d). To evaluate the effects of MMP-2 on miR-200a-mediated cell invasion, *MMP-2* was knocked down in T24T(miR-200a) cells (Fig. 2e). As shown in Fig. 2f, g, depletion of MMP-2 in T24T(miR-200a) cells significantly reversed miR-200a-enhanced cell invasion. Taken together, these results indicate that MMP-2 acts as an miR-200a downstream effector and plays a key role in BC cell invasion.

### miR-200a overexpression specifically resulted in JNK2 induction, in turn leading to c-Jun phosphorylation, and its regulated *MMP-2* transcription

**Fig. 3:**
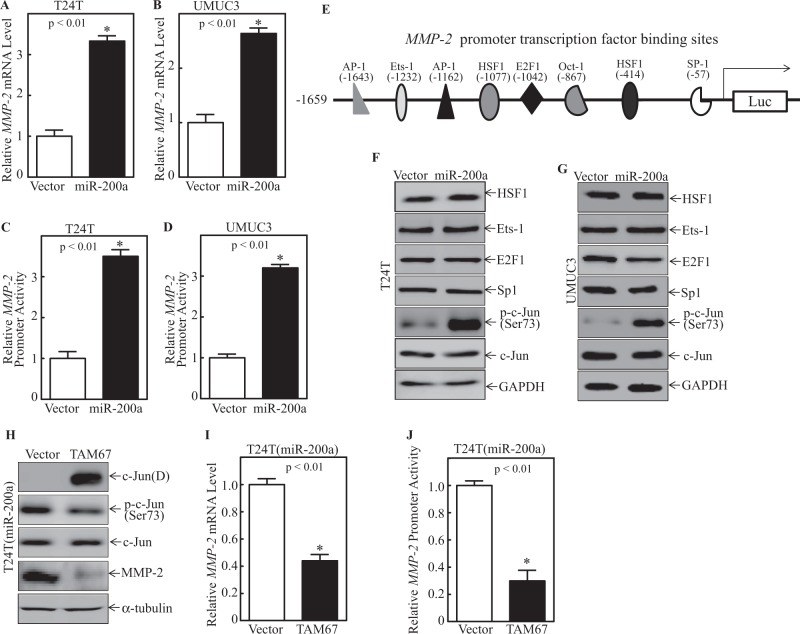
miR-200a overexpression resulted in C-Jun phosphorylation, and its regulated MMP-2 transcription and protein expression. **a**, **b** Total RNA was extracted from cells and *MMP-2* mRNA levels evaluated using real-time PCR. Wild-type *MMP-2* promoter-driven luciferase reporter was co-transfected together with pRL-TK into T24T(Vector) and T24T(miR-200a) cells (**c**), or UMUC3(Vector) and UMUC3(miR-200a) cells (**d**). After 24 h of transfection, luciferase activity was evaluated. TK was used as an internal control. Results presented as *MMP-2* promoter activity relative to control vector transfectant. Each bar represents means ± SD of three independent experiments. **e** Potential transcription factor binding sites in human *MMP-2* promoter region. Extracts obtained from T24T(Vector) vs. T24T(miR-200a) (**f**) or UMUC3(Vector) vs. UMUC3(miR-200a) (**g**) cells were analyzed for activation/expression of transcription factors indicated. **h** TAM67 and vector control were stably transfected into T24T(miR-200a) cells and transfectants were identified by western blot. **i** TAM67 and its vector control were stably transfected into T24T(miR-200a) cells; total RNA was extracted, and *MMP-2* mRNA levels were evaluated using real-time PCR). Bars represent means ± SD from three independent experiments. **j** Wild-type *MMP-2* promoter-driven luciferase reporter was co-transfected together with pRL-TK into T24T(miR-200a/Vector) and T24T(miR-200a/TAM67) cells. After 24 h of transfection, cells were extracted for determination of luciferase activity. Results were presented as *MMP-2* promoter activity relative to vector control transfectants. Each bar represents means ± SD from three independent experiments. *p < 0.01

To investigate whether miR-200a promoted the MMP-2 expression at the transcriptional level, *MMP-2* mRNA abundance was evaluated in T24T(miR-200a) *vs*. T24T(Vector) and UMUC3(miR-200a) *vs*. UMUC3(Vector) cells. *MMP-2* mRNA levels were significantly increased in both T24T and UMUC3 cells overexpressing miR-200a in comparison to those in scramble controls (Fig. 3a, b). Further analyses using *MMP-2* promoter-driven luciferase reporter indicated that overexpression of miR-200a increased *MMP-2* promoter activity (Fig. 3c, d). These results indicate that miR-200a upregulates MMP-2 at the mRNA transcription level, and in turn specifically promoting human BC invasion.

Bioinformatics analysis of *MMP-2* promoter identified multiple binding sites for various transcription factor(s) (Fig. 3e). Previous studies have also reported that transcription factors, including AP-1 [30], Ets-1 [31], HSF1 [29], and Sp1 [32], are involved in initiating MMP-2 transcription in different experimental systems. The protein levels of HSF1, Ets-1, E2F1, Sp1, and c-Jun, were assessed in miR-200a overexpressing T24T and UMUC3 cells, but no change was found at the expression levels in any of these transcription factors (Fig. 3f, g). However, phosphorylation of c-Jun at Ser73 was markedly increased by miR-200a overexpression in both T24T and UMUC3 (Fig. 3f, g), suggesting that c-Jun activation may be induced by miR-200a and subsequently contributed to MMP-2 transactivation. To test whether c-Jun activation was a key downstream event responsible for miR-200a-induced MMP-2 transcription, TAM67, a dominant-negative mutant of c-Jun, was introduced into the T24T(miR-200a) cells. As shown in Fig. 3h–j, ectopic expression of TAM67 in T24T(miR-200a) cells not only blocked MMP-2 protein expression, but also significantly inhibited *MMP-2* mRNA expression and promoter activity. These results clearly demonstrate that c-Jun activation is essential for *MMP-2* promoter transcriptional activation and protein induction by miR-200a overexpression.

**Fig. 4:**
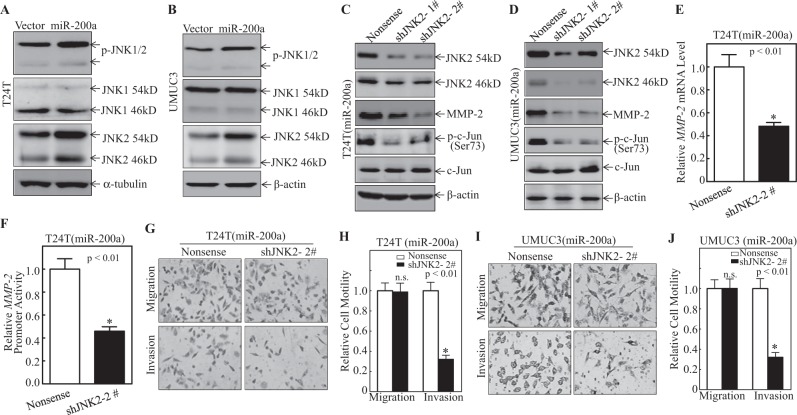
miR-200a overexpression specifically resulted in JNK2 induction, in turn leading to c-Jun phosphorylation and MMP-2 transcription. **a**–**d** Cell extracts obtained from the indicated transfectants were subjected to western blot for determination of protein expression. β-Actin or α-tubulin were used as protein loading control. **e** shJNK2 and Nonsense were stably transfected into T24T(miR-200a) cells and total RNA was extracted; *MMP-2* mRNA levels in cells were evaluated using real-time PCR. Bars represent means ± SD from three independent experiments. **f** Wild-type *MMP-2* promoter-driven luciferase reporter was co-transfected together with pRL-TK into T24T(miR-200a/nonsense) and T24T(miR-200a/shJNK2) cells. After 24 h of transfection, transfectants were used to evaluate for luciferase activity. Results presented as *MMP-2* promoter activity relative to vector control transfectants. Each bar represents means ± SD from three independent experiments. Invasion abilities of T24T(miR-200a/Nonsense) vs. T24T(miR-200a/shJNK2) cells and UMUC3 (miR-200a/Nonsense) vs. UMUC3 (miR-200a/shJNK2) cells were evaluated using invasion chamber (**g**, **i**); relative invasion ability was plotted (**h**, **j**). Bars represent means ± SD from three independent experiments. *Significant difference (*p* < 0.01)

JNK1 and JNK2, two important regulators of c-Jun activation, were next analyzed in miR-200 overexpressing cells. As shown in Fig. 4a, b, no significant change of JNK1 protein was observed in both the T24T(miR-200a) and UMUC3(miR-200a) cells as compared with the scramble vector controls. In contrast, the expression of JNK2 was greatly increased in both T24T(miR-200a) and UMUC3(miR-200a) cells under the same experimental conditions (Fig. 4a, b), suggesting a potential role of JNK2 in c-Jun activation. Moreover, knockdown of JNK2 in T24T(miR-200a) and UMUC3(miR-200a) cells diminished c-Jun phosphorylation and MMP-2 protein expression (Fig. 4c, d), as well as significantly inhibited miR-200-induced MMP-2 transcription (Fig. 4e) and promoter activity (Fig. 4f). Consistently, cell invasion was also significantly attenuated in both cell lines (Fig. 4g–j). These results reveal that upregulation of JNK2 mediated miR-200a-induced c-Jun phosphorylation, MMP-2 expression and cell invasion.

### miR-200a overexpression induced JNK2 expression via promoting JNK2 protein translation

**Fig. 5:**
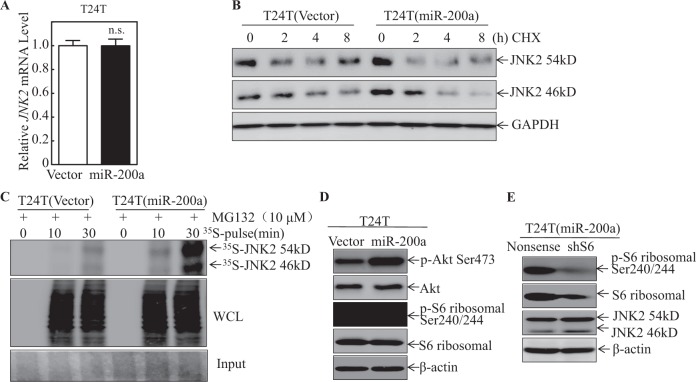
miR-200a promoted JNK2 protein translation in human BC cells. **a** Total RNA was extracted from the cells and *JNK2* mRNA levels were evaluated using real-time PCR. **b** Indicated cells were treated with cycloheximide (CHX) for denoted times and then cell extracts were subjected to western blot for determination of JNK2 protein degradation. β-Actin was used as protein loading control. **c** After treatment with MG132 (10 μM) for 30 min, newly-synthesized JNK2 protein in T24T(Vector) and T24T(miR-200a) cells was monitored by pulse assay using [^35^S]-labeled methionine/cysteine. WCL indicates whole cell lysate. Coomassie blue staining was used for protein loading control. **d** Indicated stable transfectants, T24T(Vector) and T24T(miR-200a) were used to analyze the indicated protein levels using western blot. Results shown are representative of three independent experiments. **e** shS6 and Nonsense control were stably transfected into T24T(miR-200a) cells and transfectants were identified by western blot. β-Actin was used as protein loading control. Results shown are representative of three independent experiments

To understand mechanisms underlying miR-200a-induced JNK2 upregulation, real-time PCR was performed to compare the expression levels of *JNK2* mRNA in T24T(Vector) and T24T(miR-200a) cells. The results showed that there was no observable difference in *JNK2* mRNA levels in T24T(miR-200a) cells vs. scramble vector transfectants (Fig. 5a). Thus, the potential effect of miR-200a on JNK2 protein degradation was evaluated in T24T(miR-200a) and T24T(Vector) cells. In the presence of CHX, a protein synthesis inhibitor, JNK2 protein (both 46 kD and 54 kD) exhibited a slightly accelerated protein degradation in cells overexpressing miR-200a (Fig. 5b), suggesting protein stability is unlikely the cause of increased JNK2 protein level by miR-200a. Therefore, we evaluated the possibility that miR-200a might promote JNK2 protein translation using short-term [^35^S]-methionine/cysteine pulse-labeling assays. As shown in Fig. 5c, after pretreatment of proteasome inhibitor MG132 for 4 h, incorporation of [^35^S]-methionine/cysteine into newly-synthesized JNK2 proteins was remarkably increased over time in the T24T(miR-200a) cells in comparison to that in the T24T(Vector) cells. This indicates that miR-200a overexpression promotes the synthesis of JNK2 protein by increase in JNK2 new protein translation.

To further investigate how miR-200a could affect JNK2 protein translation, the AKT/S6 ribosomal protein pathway as examined, as ribosomal protein S6 has an important role in regulating protein translation [33]. Specifically, AKT/S6 ribosomal protein activation was assessed in cells that overexpressed miR-200a. The results showed this overexpression caused an increase in S6 ribosomal protein phosphorylation at Ser240/244 in the T24T(miR-200a) cells as compared with in T24T(Vector) cells. Consistently, the phosphorylation of AKT at Ser473 showed similar changes between the T24T(miR-200a) and T24T(Vector) cells (Fig. 5d). However, knockdown of S6 led to no observed change in JNK2 protein in T24T(miR-200a/shS6) in comparison to T24T(miR-200a/nonsense) cells (Fig. 5e). The upshot of these results reveals that the AKT/S6 ribosomal protein pathway was not involved in the earlier-noted miR-200a upregulation of JNK2 protein translation.

### miR-200a inhibited miR-16, causing a reduction of miR-16 binding to the 3′-UTR of *JNK2* mRNA and consequently increased JNK2 protein translation

**Fig. 6:**
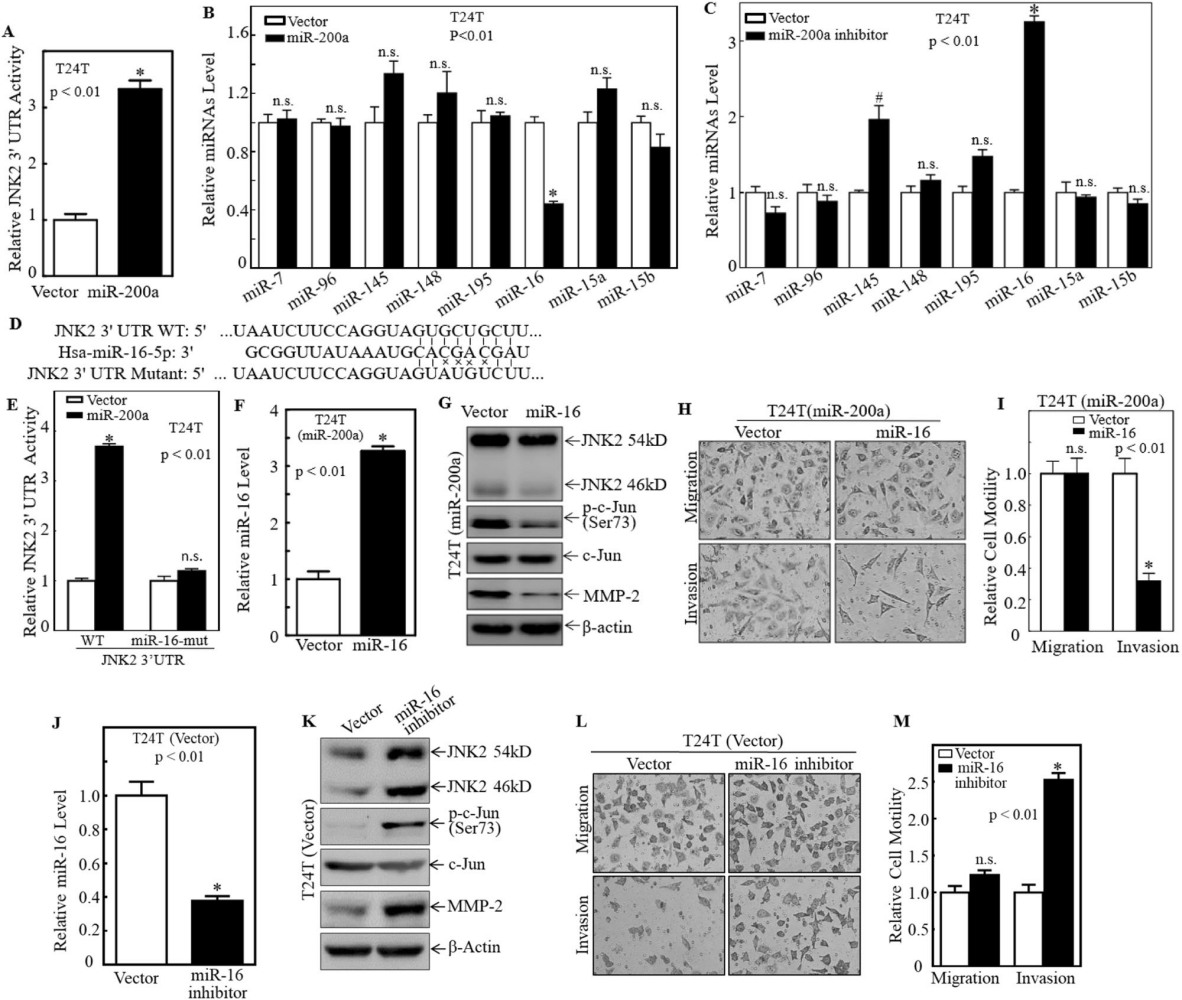
miR-200a inhibited miR-16 expression, therefore reducing miR-16 binding to 3′-UTR of JNK2 mRNA, and increasing JNK2 protein translation. **a** *JNK2* 3′-UTR activity was evaluated by transfection of *JNK2* 3′-UTR-driven luciferase reporter along with pRL-TK into transfectants as indicated. Bars represent means ± SD from three independent experiments. **b** miRNA expression levels were evaluated by real-time PCR in T24T(Vector) vs. T24T(miR-200a) cells. Results were normalized to U6. **c** miRNA expression levels were evaluated by real-time PCR in T24T(Vector) vs. T24T (miR-200a inhibitor) cells. Results were normalized to U6. **d** Schematic of miR-16 binding site in JNK2 mRNA 3′-UTR region and its mutants aligned with miR-16. **e** T24T(Vector) and T24T(miR-200a) cells were co-transfected with wild-type and mutant *JNK2* 3′-UTR luciferase reporters and pRL-TK, respectively. Luciferase activity of each transfectant was evaluated and results were presented as relative *JNK2* 3′-UTR activity. **f** Real-time PCR was used to identify the miR-16 expression in T24T(miR-200a/Vector) vs. T24T (miR-200a/ miR-16) cells. Bars represents means ± SD from three independent experiments. **g** Extracts from T24T (miR-200a/ Vector) vs. T24T (miR-200a/ miR-16) cells were used to evaluate effect of miR-16 on expression of JNK1, JNK2, p-c-Jun, c-Jun, and MMP-2 by western blot. β-Actin was used as protein loading control. Results shown are representative of three independent experiments. Invasion abilities of T24T(miR-200a/Vector) and T24T(miR-200a/miR-16) cell were evaluated using Invasion Chambers (**h**); and the relative invasion ability was plotted (**i**). Bars represent means ± SD from three independent experiments. **j** Real-time PCR was used to identify the miR-16 expression in T24T(Vector) vs. T24T (miR-16 inhibitor) cells. Bars represents means ± SD from three independent experiments. **k** Extracts from T24T(Vector) vs. T24T (miR-16 inhibitor) cells were used to evaluate effect of miR-16 on expression of JNK1, JNK2, p-c-Jun, c-Jun, and MMP-2 by western blot. β-Actin was used as protein loading control. Results shown are representative of three independent experiments. Invasion abilities of T24T(Vector) and T24T (miR-16 inhibitor) cell were evaluated using invasion chambers (**l**); and the relative invasion ability was plotted (**m**). Bars represent means ± SD from three independent experiments. *p < 0.01, ^#^*p* < 0.05

To explore the mechanisms leading to induction of JNK2 protein translation by miR-200a overexpression, the *JNK2* 3′-UTR-driven luciferase reporter was introduced into T24T(miR-200a) and T24T(Vector) cells. The results showed that ectopic expression of miR-200a significantly increased *JNK2* 3′-UTR luciferase reporter transcriptional activity (Fig. 6a), suggesting that miRNAs might be involved in JNK2 protein translation upon miR-200a overexpression. The “targetscan.org” was next used to analyze key potential miRNA binding sites in the *JNK2* 3′-UTR luciferase reporter and it indicated that JNK2 3′-UTR contained multiple binding sites for miRNAs, including miR-7, miR-96, miR-145, miR-148, miR-195, miR15a, miR-15b, and miR-16, in JNK2 mRNA 3′-UTR region (Table S1). Real-time PCR was further performed to evaluate the relative expression levels of these miRNAs in T24T(miR-200a) vs. T24T(Vector) cells as well as T24T (miR-200a inhibitor) vs. T24T(Vector) cells. As shown in Fig. 6b, c, among eight analyzed miRNAs, only miR-16 was significantly decreased in T24T(miR-200a) cells (Fig. 6b), and significantly upregulated in T24T(miR-200a inhibitor) cells (Fig. 6c). Moreover, point mutations in the miR-16 binding site in the *JNK2* mRNA 3′-UTR luciferase reporter completely abolished the miR-200a-increased luciferase transcription activity, suggesting that the miR-16 binding site was crucial for miR-200a promotion of *JNK2* mRNA 3′-UTR activation (Fig. 6d, e). To verify this, miR-16 was overexpressed in T24T(miR-200a) and UMUC3(miR-200a) cells (Fig. 6f and S4). The levels of JNK2, MMP-2, and p-c-Jun were decreased in miR-16 overexpressing cells (Fig. 6g and S4), which coincided with dramatically reduced cell invasion in these cells (Fig. 6h, i). Consistently, the inhibition of the miR-16 expression in T24T cells using miR-16 inhibitor elevated protein levels of JNK2 and MMP-2, enhanced c-Jun phosphorylation (Fig. 6j, k), and increased cell invasion (Fig. 6l, m), which are quite similar to those observed in miR-200a overexpression T24T cells (Fig. 1l, m). Therefore, it is likely that miR-200a overexpression inhibits miR-16 abundance, which results in an increased JNK2 protein translation and subsequently upregulating c-Jun phosphorylation and MMP-2 transcription, as well as BC cell invasion.

### miR-200a overexpression inhibited Dicer protein expression, and in turn attenuated miR-16 maturation and BC invasion

**Fig. 7:**
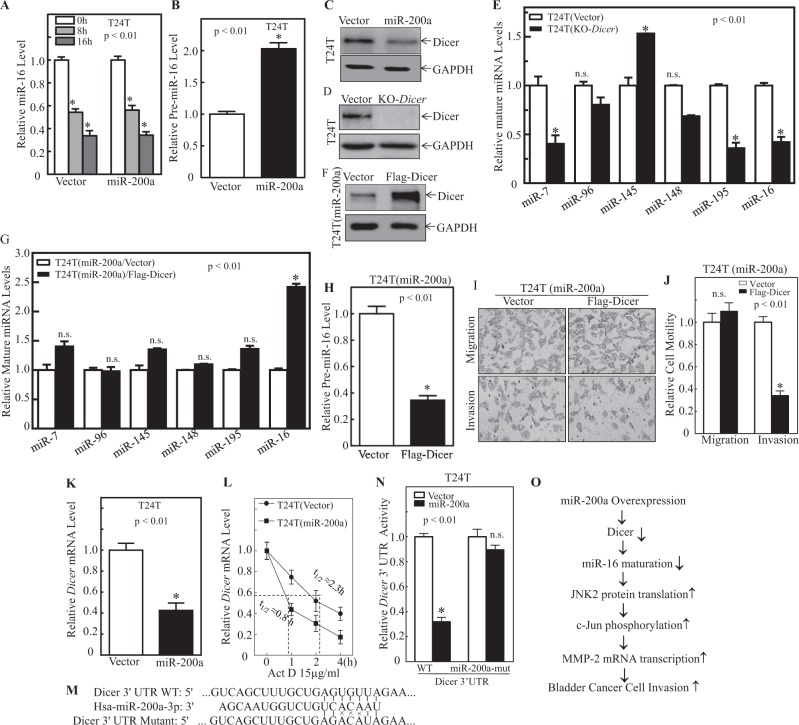
miR-200a inhibited Dicer expression, therefore attenuating miR-16 maturation. **a** Relative miR-16 stability was evaluated by real-time PCR in T24T(Vector) vs. T24T(miR-200a) cells after treatment with Act D for indicated times. **b** Pre-miR-16 expression was evaluated in T24T(Vector) and T24T(miR-200a) cells using real-time PCR. Bars represent means ± SD from three independent experiments. **c** Extracts from T24T(Vector) and T24T(miR-200a) cells were used to evaluate effect of miR-200a on Dicer expression; GAPDH used as a protein loading control. **d** Extracts from T24T(Vector) and T24T(KO-Dicer) cells were used to evaluate Dicer expression. GAPDH used as a protein loading control. **e** miRNA expression levels were evaluated by real-time PCR in T24T(Vector) vs. T24T(KO-Dicer) cells. Results were normalized to U6. **f** Extracts from T24T (miR-200a/Vector) and T24T(miR-200a/flag-Dicer) cells were used to evaluate Dicer expression. GAPDH used as a protein loading control. **g** miRNA expression levels were evaluated by real-time PCR in T24T(miR-200a/Vector) and T24T(miR-200a/flag-Dicer) cells. Results were normalized to U6. **h** Relative pre-miR-16 expression level were evaluated in 24T (miR-200a/Vector) and T24T(miR-200a/flag-Dicer) cells. Bars present means ± SD from three independent experiments. T24T(miR-200a/Vector) and T24T (miR-200a/flag-Dicer) cell invasion abilities were evaluated using invasion chambers (**i**); and the relative invasion ability was plotted (**j**). **k** Relative *Dicer* mRNA level was analyzed in T24T(Vector) and T24T(miR-200a) cells. **l** Relative *Dicer* mRNA stability was evaluated by real-time PCR in T24T(Vector) vs. T24T(miR-200a) cells after treatment with Act D for indicated times. **m** Schematic of miR-200 binding site in *Dicer* mRNA 3′-UTR region and its mutants aligned with miR-200a. **n** T24T(Vector) and T24T(miR-200a) cells were co-transfected with wild-type and mutant *Dicer* 3′-UTR luciferase reporters and pRL-TK, respectively. Luciferase activity of each transfectant was evaluated and results were presented as relative *Dicer* 3′-UTR activity. *Significant difference in *Dicer* 3′-UTR activity (*p* *<* 0.01). **o** The schematic summary of molecular mechanisms underlying miR-200a overexpression in the promotion of human BC cell invasion

To further understand why miR-16 abundance was decreased, potential effects of miR-200a on the regulation of miR-16 stability was assessed. No significant change of miR-16 stability was observed in T24(miR-200a) and T24T(Vector) cells (Fig. 7a). Analyses of pre-miR-16 levels revealed a marked increase of pre-miR-16 in T24T(miR-200a) in comparison to T24T(Vector) (Fig. 7b). This suggested that dysregulation of miR-200a might affect miR-16 maturation in human BC cells.

Dicer plays a role in processing miRNA maturation. We therefore evaluated the protein level of Dicer in miR-200a overexpressing cell. As shown in Fig. 7c, Dicer expression was reduced in the T24T(miR-200a) cells as compared with that in T24T(Vector) cells. To test whether reduced Dicer in T24T(miR-200a) cells contributed to impaired miR-16 maturation, we depleted Dicer in T24T cells using CRISPR/Cas9 (Fig. 7d), and analyzed the levels of several mature miRNAs. As shown in Fig. 7e, only three out of six tested miRNAs, including miR-7, miR-195, and miR-16, were significantly decreased in their mature miRNA levels. miR-96 and miR-148 were not significantly altered by Dicer knockout, whereas miR-145 levels were increased in Dicer KO cells. Moreover, ectopically expressed Flag-tagged Dicer in T24T(miR-200a) cells only led to a remarkable increase of miR-16 expression, whereas other miRNAs were not significantly affected by Dicer overexpression (Fig. 7f, g). Consistently, pre-miR-16 levels were significantly decreased in the T24(miR-200a/flag-Dicer) cells in comparison to scramble T24T(miR-200a/Vector) cells (Fig. 7h). Cell invasion assay showed that invasion ability was impaired in T24T(miR-200a/flag-Dicer) cells as compared with that in T24T(miR-200a/Vector) cells (Fig. 7i, j). These results suggested that the dysregulation of miR-200a in BC cells downregulated Dicer and miR-16 expression, which subsequently lead to c-Jun activation as well as increased MMP-2 expression and cell invasion. It was noted that maturation of some miRNAs was not affected by depletion of Dicer (Fig. 7e), suggesting that a Dicer-independent mechanism may modulate other miRNA maturation. This notion is consistent with the findings in previous published studies [34, 35]. In addition, our results also showed that despite a few miRNA maturations were affected in Dicer knockout cells, only miR-16 was induced by overexpressing Dicer (Fig. 7e, g). These results suggested that there might be differential thresholds of Dicer for various miRNAs maturation. Although we don’t know the molecular mechanisms underlying this observation, we anticipate that miR-16 may be more sensitive to the changes of the cellular level of Dicer. We are currently working this very interesting observation and hope that we could have more results on this study in our near future publication.

To understand the mechanism underlying reduced Dicer protein by miR-200a overexpression, we analyzed *Dicer* mRNA levels using real-time RT-PCR. As shown in Fig. 7k, overexpression of miR-200a in T24T cells resulted in a significant decrease in *Dicer* mRNA levels. Next, mRNA stability assay revealed accelerated degradation of *Dicer* mRNA in T24T(miR-200a) *vs.* T24T(Vector) cells (Fig. 7l). Moreover, miR-200a overexpression significantly attenuated *Dicer* mRNA 3′-UTR reporter transcription activity, while mutation of miR-200a binding site in *Dicer* mRNA 3′-UTR luciferase reporter resulted in a loss of this reporter response to miR-200a overexpression (Fig. 7m, n), revealing that miR-200a inhibition of *Dicer* mRNA 3′-UTR activity was through its binding site in *Dicer* mRNA 3′-UTR. Given that Dicer has been reported to be downregulated by oxidative stress [36], we also explored the possible involvement of oxidative stress in miR-200a overexpressing cells. Superoxide dismutase 2 (SOD2), an important mitochondria enzyme to convert superoxide to hydrogen peroxide, was reduced in T24T(miR-200) cells (Fig. S5), suggesting an increased oxidative stress in these cells. Collectively, our results indicate that overexpressed miR-200a binds to its binding site in 3′-UTR of *Dicer* mRNA and inhibits Dicer protein translation as well as miR-16 maturation. The latter subsequently results in the upregulation of JNK2 protein followed by c-Jun activation and MMP-2 transcription, which eventually leads to enhanced BC cell invasion as illustrated in Fig. 7o.

## Discussion

It is urgent to define mechanisms that underlie BC cell invasive capacities so that researchers can define new therapeutic approaches to treat this malignant disease. There are several reports that miR-200a may have an oncogenic role in cancer cells [37, 38]. It has been shown that miR-200a inhibits cell migration and invasion, as well as EMT in hepatocellular carcinoma [39, 40]. However, any role of miR-200a in BC cell migration/invasion has not been elucidated to the best of our knowledge. The present study, using gain (overexpression) and loss (knockdown) strategies, identified an oncogenic function for miR-200a in promoting cancer invasion in human BC cells.

Dicer plays an important role in processes of miRNA maturation [41] and its expression has been reported to be upregulated in prostate adenocarcinoma [14], but downregulated in ovarian [15] and lung cancers [16]. Interestingly, both low and high expression levels of Dicer have been correlated with poor prognoses in cancer patients in various cancers [17]. Because Dicer can catalyze biosynthesis of miRNA and siRNA, in turn regulating the expression of numerous genes, expression of a *Dicer* gene itself is likely to be highly regulated [18, 19]. Some studies indicate that discrepancies in dysregulation of Dicer expression among various tumor types could be attributed to tissue-specific differences/degree of aggressiveness of the cancer [20, 21]. Although Dicer expression has been reported to be downregulated in BCs [22], and downregulated Dicer could suppress cell proliferation in BC T24 cells [23], we know nothing about the function of Dicer in BC cell migration and invasion. The present study found that Dicer protein expression was significantly decreased in BC cells that overexpressed miR-200a. Moreover, miR-16 expression was also increased significantly, whereas pre-miRNA levels were dramatically decreased after overexpression of Dicer in T24T(miR-200a) cells in comparison to scramble transfectants. These results suggest that Dicer is a miR-200a downstream mediator being responsible for promotion of miR-16 maturation in human BC cells.

While miR-16 has been shown to regulate proliferation and apoptosis in many types of cancers, its potential function in BC is not understood. Jiang et al. have reported that miR-16 expression was downregulated in BCs in comparison with the adjacent normal tissues; and miR-16 could inhibit BC cell proliferation by targeting cyclin D1 in TCHu-1 cells [42]. miR-16 also acts as a considerable modulator of inducible apoptosis of BC cells by causing downregulation of COX-2 expression [43]. It was recently shown that miR-16 could downregulate COX-2 expression via deactivation of NF-κB signaling pathway upon puerarin treatment in T24 cells. Interestingly, the present study showed that miR-16 maturation was inhibited in T24T cells that overexpressed miR-200a. Thus, based on the findings here, the overexpression of miR-200a leads to Dicer inhibition, and in turn impairs miR-16 maturation and this likely impacts on the invasion of the BC cells.

Cell migration is an integral property of many types of cells and critical for normal development, immune responses, and disease processes like cancer [44]. Migration is also a key to metastatic potentials of any given tumor cell types. Cancer metastasis includes multiple steps such as local tumor cell invasion, entry into vasculature, cancer cell exit from circulation, and colonization at distal sites [45]. While many types of normal cells have migration abilities, tumor cells, especially malignant cells, have this ability and the capacity to be invasive [46]. Invasive abilities of cancer cells are related to migration and many other factors, including expressions of MMP-2, MMP-9, Rac1, Rho A, and SOD2 [26]. Elucidating underlying mechanisms about the underlying bases for migration/invasion of BC cells is important if one wants to develop strategies/novel drugs for the treatment of BC. Hydration of the extracellular matrix (ECM) is an important process that allows cancer cell invasion and metastasis through the secretion of enzymes such as MMP-2 and MMP-9. Of the aforementioned proteins in [22], MMP-2 and MMP-9 play critical roles in the mediation of ECM degradation. The MMP-2 and MMP-9 are two major MMPs that play a critical role in ECM degradation of the main component of the ECM, which is called hydrolyzing type IV collagen [47]. MMP-2 and MMP-9 were overexpressed in many cancers, and this was correlated with increases in cancer cell invasion and numbers/levels of metastases in many types of cancer [48–50]. Interestingly, some earlier studies from our laboratories have reported that the overexpression of MMP-2 is also crucial for human BC invasion [26], while inhibiting MMP-2 expression by anticancer agent ISO significantly attenuates both BC invasion in vitro and highly invasive BC formation in vivo [27]. In the present study, it was found that MMP-2 expression levels correlated well with miR-200a overexpression and BC cell invasion. The present experiments further indicated that c-Jun phosphorylation was increased in miR-200a overexpressing cells. Through binding to the *MMP-2* promoter, p-c-Jun upregulated *MMP-2* transcription and expression, further promoting the invasion of the miR-200 overexpressing BC cells.

To further clarify the role of the changes in c-Jun activation as they pertain to changes in BC cell functions, levels of JNK1 and JNK2 were analyzed. The results indicated that the expression of JNK2, but not JNK1, was increased by miR-200 overexpression. This indicated that JNK2 was likely responsible for the upregulation of p-c-Jun by miR-200a. By measuring *JNK2* mRNA levels in T24T(Vector) and T24T(miR-200a), we demonstrated that JNK2 was upregulated at the protein translation level. The [^35^S]-labeled methionine and cysteine for new protein synthesis studies here clearly revealed that miR-200a was able to increase JNK2 translation, which is a novel mechanism specifically leading to JNK2 protein expression without affecting its protein phosphorylation, and further resulting in c-Jun phosphorylation and activation. Taken together, these results demonstrate that miR-200a could contribute to an upregulated expression of JNK2 that, in turn, causes an increase in c-Jun phosphorylation, consequently promoting MMP-2 transcription and its enhancement of BC cell invasion.

In summary, this study showed that miR-200a was overexpressed in BCs and promoted BC invasion, at least in part, by causing an upregulation of the MMP-2 expression. This study also proved that p-c-Jun had a critical role in regulating *MMP-2* transcription in the experimental BC cell model here, and that miR-200a downregulated Dicer protein expression and inhibited miR-16 maturation. The latter outcome led to increases in JNK2 protein translation and c-Jun phosphorylation, thereby promoting BC cell invasion. The present study provided significant insights into the understanding of how miR-200a might impact on BC cell invasion, and ultimately invasive tumor development.

## Material and methods

### Reagents, cell lines, and cell culture

The normal bladder cell line UROtsa and human BC cell lines T24T, UMUC3 were described in previous studies [51–54]. For the details of reagents, cell lines, and cell culture, see the Supplement of “Materials and Methods”.

### Cell transfection and western blot

Cell transfections were performed with PolyJet^TM^ DNA in vitro Transfection Reagent (# SL100688, SignaGen Laboratories, Rockville, MD, USA). Cell extracts were subjected to western blot analysis as described previously [55–57]. For more details, please see the Supplement of “Materials and Methods”.

### BBN-induced highly invasive BC in mice

C57BL/6J mice (males, 3–4 weeks old) were purchased from (Jackson Laboratory, Bar Harbor, ME, USA). All mice were randomly allocated into two groups and then exposed to BBN for invasive BC induction as described in the Supplement of “Materials and Methods”.

### Human BC tissue samples

A total of 17 pairs of primary BC samples and their paired adjacent normal bladder tissues were obtained from patients who underwent radical cystectomy between 2012 and 2013. For more details, please see the Supplement of “Materials and Methods”.

### Quantitative real-time PCR and luciferase reporter assay

Total RNA from various cells/sample were extracted using TRIzol (Invitrogen, Grand Island, NY) [29]. Total miRNA was extracted using miRNeasy Mini Kit (Qiagen, Valencia, CA). Luciferase reporter assays were performed as described previously [52, 58]. More details of real-time PCR and luciferase reporter assay were provided in the Supplement of “Materials and Methods”.

### [^35^S]-Methionine pulse assays

[^35^S]-methionine/cysteine was employed to determine JNK2 protein translation as described in the Supplement of “Materials and Methods”.

### Cell proliferation assay and invasion assay

The cell proliferation was measured using CellTiter-Glo Luminescent Cell Viability Assay kit (Promega, Madison, WI, USA). The invasion assay was performed using transwell assay kit purchased from BD Biosciences (Bedford, MA, USA). For more details, please see the Supplement of “Materials and Methods”.

### Statistical analysis

All data are presented as means (triplicate assays) ± SD. A Student’s *t* test was employed to determine the significance of differences between the various groups. Differences were considered significant at *p* *<* 0.05. All statistical analyses were performed using Prism 5.0 Software (GraphPad, San Diego, CA, USA).

## Supplementary information

Supplemental Material

## Data Availability

Datasets supporting the conclusions of this article are included within the article.
