# Supplementary material for: Overexpressed miR-200a promotes bladder cancer invasion through direct regulating Dicer/miR-16/JNK2/MMP-2 axis
Source: Oncogene. 2019 Nov 26;39(9):1983–96. doi: 10.1038/s41388-019-1120-z (PMC7044116; doi:10.1038/s41388-019-1120-z)
Supplement: Supplementary file 1 — Supplemental Material [file 41388_2019_1120_MOESM1_ESM.docx]

***Supplementary Material:***

**Over-expressed miR-200a Promotes Bladder Cancer Invasion Through Direct Regulating Dicer/miR-16/JNK2/MMP-2 Axis**

Rui Yang^#^, Jiheng Xu^#^, Xiaohui Hua^#^, Zhongxian Tian, Qipeng Xie, Jingxia Li, Guosong Jiang, Mitchell Cohen, Hong Sun, and Chuanshu Huang^*^

^#^These authors contributed equally to this work.

*** Corresponding author:** Chuanshu Huang, M.D. & Ph.D. Department of Environmental Medicine, New York University School of Medicine, 341 East 25th Street, New York, New York 10010; Tel: 646-754-9457; Fax: 646-754-9471; E-mail: [Chuanshu.huang@nyulangone.org](mailto:Chuanshu.huang@nyulangone.org).


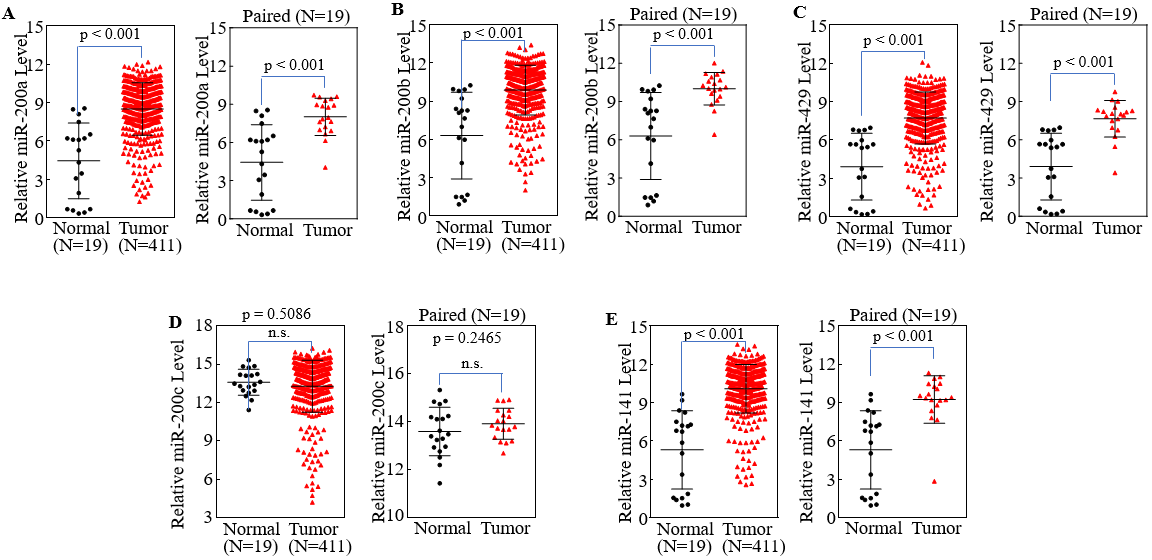


**Figure S1.** Comparison of miR-200 family expression in human bladder tumor *vs*. normal bladder tissues by RNA-seq (Illumina-Hiseq) based on TCGA database.


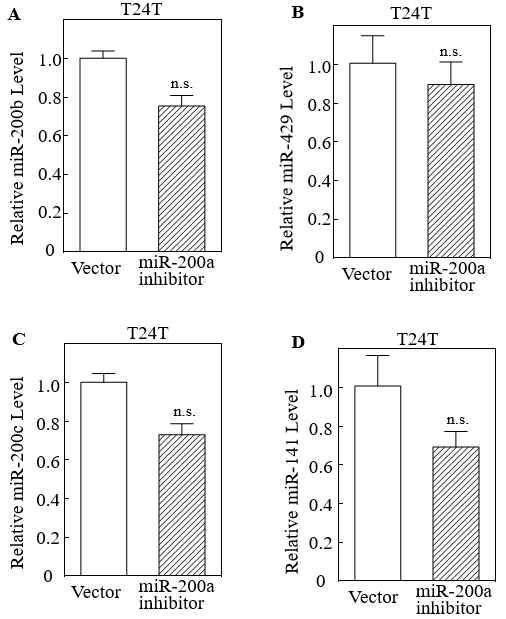


**Figure S2.** Real-time PCR was used to evaluate the effect of miR-200a inhibitor on other four members of miR-200 family.


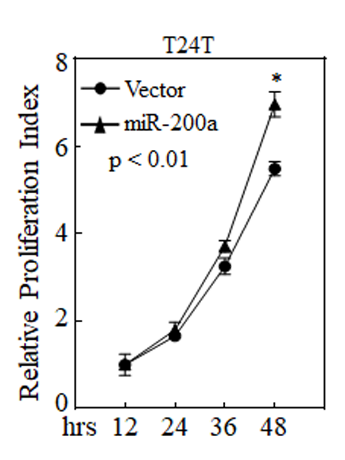


**Figure S3.** The cell proliferation was analyzed using CellTiter-Glo Luminescent Cell Viability Assay kit with a luminometer in T24T(Vector) vs. T24T(miR-200a).


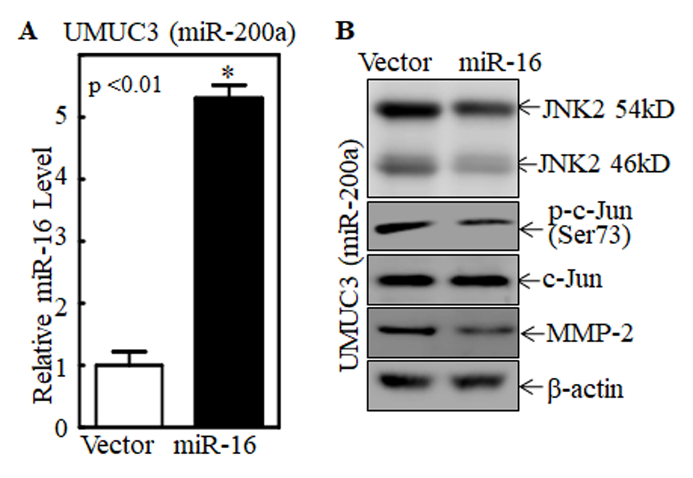


**Figure S4. (A)** Real-time PCR was used to identify miR-16 expression in UMUC3(miR-200a/Vector) *vs.* UMUC3 (miR-200a/ miR-16) cells. Bars represents means ± SD from three independent experiments. *Significant difference (p < 0.01). **(B)** Extracts from UMUC3(miR-200a/Vector) *vs.* UMUC3(miR-200a/miR-16) cells were used to evaluate effect of miR-16 on expression of JNK1, JNK2, p-c-Jun, c-Jun, MMP-2 by WB. β-actin was used as protein loading control. Results shown are representative of three independent experiments.


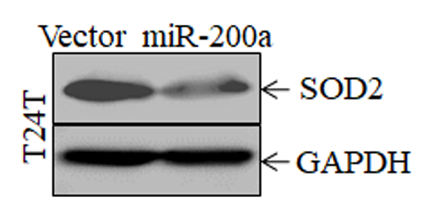


**Figure S5.** Extracts from T24T(Vector) and T24T(miR-200a) cells were used to evaluate effect of miR-200a on SOD expression; GAPDH used as a protein loading control.

**Table S1.** The potential miRNAs binding sites in JNK2 mRNA 3’UTR region


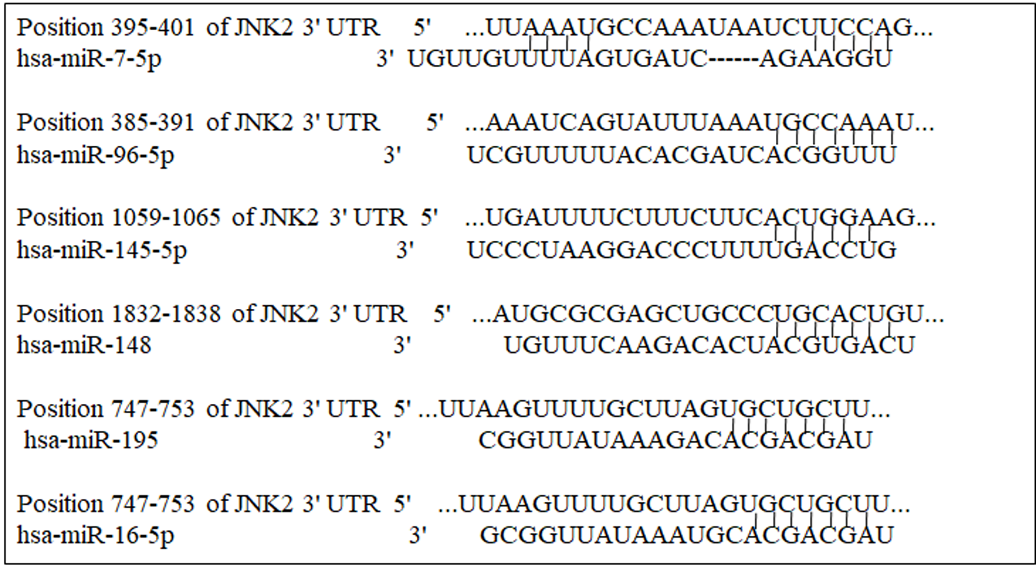


**Supplement of Materials and Methods**

***Plasmids, antibodies, and reagents***

A human *MMP-2* promoter luciferase reporter was a gift from Dr. Etty N. Benveniste (Department of Cell Biology, University of Alabama at Birmingham) [1]. Dicer knock out (#16656) plasmid as well as over-expression of miR-200a (#35533), miR-16 (#51382), and Flag-tagged Dicer (#19881) plasmids were obtained from Addgene (Cambridge, MA, USA). The short hairpin RNA constructs specific to JNK2(shJNK2), S6(shS6), MMP-2 (shMMP-2) (# RHS4533) and their scramble nonsense control constructs were purchased from Open Biosystem (Pittsburgh, PA, USA). The miR-200a inhibitor (#HmiR-AN0302-AM03-B) and miR-16 inhibitor (#HmiR-AN0227-AM03) were purchased from GeneCopia (Rockville, MD, USA). The TAM67 plasmid, a well-characterized dominant-negative c-Jun mutant, was described in our previous studies [2]. The *JNK2* 3'-UTR luciferase reporter has been described previously [3]. The mutation of *JNK2* 3'-UTR luciferase at the miR-16 binding site was made by point mutation using the following primers: Sense: 5'-ATC TTC CAG GTA GTG CTG CTT CTG AAG TTA TCT-3' antisense: 5'-AGA TAA CTT CAG AAG CAG CAC TAC CTG GAA GAT -3'. The *Dicer* 3'-UTR fragment was amplified and cloned into pMIR-Report vector (Ambion, Austin, TX, USA) at the XhoI and HindIII (New England Biolabs (NEB), Beverly, MA, USA) sites, respectively. The mutation of *Dicer* 3'-UTR luciferase at the miR-200a binding site was made by point mutation using the following primers: Sense: 5'-GTC AGC TTT GCT TAG ACA TAG AAG GCC ATG GAG-3' antisense: 5'-CTC CAT GGC CTT CTA TGT CTA AGC AAA GCT GAC-3'.

Antibodies specific against p-c-Jun (Ser73) (3270s), c-Jun (9165s), p-JNK1/2, JNK1, JNK2 (9258s), p-Akt (Ser473) (4060s), Akt (4691s), p-S6 ribosomal (Ser240/244), S6 ribosomal (2217s), and Dicer (5362P) were purchased from Cell Signaling Technology (Beverly, MA, USA). Antibodies against MMP-2 (sc-10736), c-Jun(D) (sc-44), HSF1, ETS-1 (sc-55581), E2F1 (sc-251), Sp1(sc-14027) and GAPDH (sc-25778) were bought from Santa Cruz Biologics (Santa Cruz, CA, USA). Antibody against α-tubulin (66031-1-Ig) and β-Actin (66009-1-Ig) were purchased from Proteintech Group Inc. (Chicago, IL, USA).

BBN (#B0938) was purchased from TCI AMERICAN (Cambridge, MA, USA). Proteasome inhibitor MG132 (#S2619) was bought from Selleckchem (Houston, TX, USA); Actinomycin D (Act D) (#sc-200906) and Protein synthesis inhibitor cycloheximide (CHX) (CAS 66-81-9) (#sc-3508) were purchased from Santa Cruz Biologics. A dual luciferase assay kit was brought from Promega (Madison, WI, USA). TRIzol and a SuperScript First-Strand Synthesis system were acquired from Invitrogen (Grand Island, NY, USA). PolyJet DNA *in Vitro* Transfection Reagent was purchased from SignaGen Labs (Rockville, MD, USA).

***Cell lines and cell culture***

The normal bladder cell line UROtsa and human BC cell lines T24T, UMUC3 were described in previous studies [4-7]. T24T cells were maintained in 1:1 mixture of DMEM/Ham’s F12 medium. (Invitrogen, Carlsbad, CA, USA) supplemented with 5% heat-inactivated fetal bovine serum (FBS) (ATLANTA, Flowery Branch, GA, USA), a mixture solution of 2 mM L-glutamine, 100 IU penicillin and 100 μg/ml streptomycin (CORNING, 30-009-CI). UMUC3 cells were cultured in DMEM (Invitrogen, Carlsbad, CA, USA) containing 10% heat-inactivated FBS (ATLANTA, Flowery Branch, GA, USA), a mixture solution of 2 mM L-glutamine, 100 IU penicillin and 100 μg/ml streptomycin (CORNING, 30-009-CI). UROtsa cells were cultured in RPIM 1640 with 10% FBS (ATLANTA, Flowery Branch, GA, USA). All lines were subjected to DNA tests and were authenticated before/after utilization for research by Genetica DNA Laboratories (Burlington, NC, USA) using a PowerPlex16 HS System.

***Cell transfection***

Cell transfections were performed with PolyJet^TM^ DNA *in Vitro* Transfection Reagent (# SL100688, SignaGen Laboratories, Rockville, MD, USA) according to manufacturer instructions. For stable transfection, cell cultures were subjected to selection with G418 (500-1000μg/ml) (#G-418-25, GoldBio, St. Louis, MO, USA ), or puromycin (0.2–0.3μg/ml) (#P-600-100, GoldBio, St. Louis, MO, USA ), depending on the different antibiotic resistance plasmid transfected. Cells surviving antibiotic selection were pooled as stable mass transfectants.

***Western blot analysis***

The parent T24T and UMUC3 cells and their transfectants were seeded and cultured in 6-well plates until 70-80% confluence. At that point, medium was removed, and cells were cultured 0.1% FBS-containing medium for 12 hr. Thereafter, the medium was replaced with appropriate 10% FBS-DMEM or 5% FBS-DMEM-F12 (1:1) for another 12 hr. Whole-cell extracts were then prepared using cell lysis buffer (10 mM Tris-HCl ( pH 7.4) (Sigma-Aldrich, Steinheim, Germany), 1% SDS（Fisher Scientific Co., Rochester, NY, USA ）, and 1 mM Na_3_VO_4_ (Fisher Scientific Co., Rochester, NY, USA )). Cell extract protein was then determined using Nanodrop 2000(Thermo Scientific, Waltham, MA). Aliquots containing 100 µg protein were then subjected to SDS-PAGE separation/electro-transfer/Western blot analysis as described previously [8-10]. Images of the antibody-tagged protein(s) of interest were then acquired by scanning with a Typhoon FLA 7000 PhosphorImager system (GE Healthcare, Pittsburgh, PA, USA). Densitometry analysis was done with Image J software following the user guide.

***BBN-induced highly invasive BC in mice***

C57BL/6J mice (males, 3-4 wks. old) were purchased from (Jackson Laboratory, Bar Harbor, ME, USA). All mice were housed in pathogen-free facilities maintained at 25°C with a 50-60% relative humidity and a 12-hr light/dark cycle. All mice had *ad libitum* access to standard rodent chow and filtered water. After 6-8 wks. of acclimatization, mice were randomly allocated into two groups, i.e., a vehicle negative control and a BBN-treated group. In the latter, each mouse was supplied (*ad libitum* for 23 wks.) opaque bottles bearing tap water with 0.05% [v/v] BBN (TCI America, Portland, OR) and thereafter (for 23 wks.) with BBN-free water. BBN water was prepared fresh twice/week. Individual mouse consumption was recorded daily to estimate BBN intake. Negative control mice received regular tap water throughout. Mice from each group were euthanized by carbon dioxide at week 24 post-BBN exposure and subjected to necropsy to recover bladder tissues/tumors. All procedures involving the mice were conducted in compliance with guidelines set forth for ethical animal research and were approved by the New York University School of Medicine Institutional Animal Care and Use Committee.

***Human bladder cancer tissue samples***

A total of 17 pairs of primary bladder cancer samples and their paired adjacent normal bladder tissues were obtained from patients who underwent radical cystectomy between 2012 and 2013 at the Department of Urology at the Union Hospital of Tongji Medical College (Wuhan, China). All specimens were immediately snap-frozen in liquid N_2_ after surgical removal. Histological and pathological diagnoses were confirmed, and specimens classified by a certified clinical pathologist using the 2004 World Health Organization Consensus Classification and Staging System for bladder neoplasms. All specimens were obtained with appropriate informed consent from the patients; a supportive grant was obtained from the Medical Ethics Committee of China. Experiments were carried out in accordance with the Code of Ethics of the World Medical Association (Declaration of Helsinki) for experiments involving humans.

***Luciferase reporter assay***

Luciferase reporter assays were performed as described in Liang et al. (2016) [5] and Xie et al. (2016)[11]. Each luciferase reporter construct tested, together with pRL-TK vector (Promega, Madison, WI, USA), was transiently transfected into the indicated cells. After 24 hr., luciferase activity was determined using a luciferase Assay System kit (Promega, Madison, WI, USA) according to manufacturer’s protocols. All results were normalized against the internal TK signal.

***Real-time PCR for mRNA***

Total RNA from UROtsa, T24T and UMUC3 cells/sample were extracted using TRIzol (Invitrogen, Grand Island, NY) as manufacturer instructions. From each extract, aliquots of Total RNA (5 μg) were used for first-strand cDNA synthesis with oligo (dT) primer using a SuperScript First-Strand Synthesis system (Invitrogen, Grand Island, NY). The primers used in this study were : human *MMP-2* (Forward: 5'-CAA GTG GGA CAA GAA CCA GA -3', Reverse: 5'-CCA AAG TTG ATC ATG ATG TC -3'), human *JNK-2* (Forward: 5'-ATG AAG AAA CTT CAG CCA ACT GT -3', Reverse: 5'-ACA GAT CTC TGG CTT GAC TT -3') and human *GAPDH* (Forward: 5'-GAT GAT CTT GAG GCT GTT GTC -3', Reverse: 5'-CAG GGC TGC TTT TAA CTC TG-3'). Real-time PCR was then conducted following the protocol for the Fast SYBR Green Master Mix kit (Applied Biosystems, Foster City, CA, USA); the assay was performed in a 7900HT Fast Real-Time PCR System (Applied Biosystems) using the same cDNA used for RT-PCR as described in our previous publication [12].

***Quantitative Real-time PCR for miRNA***

Total miRNA was extracted using miRNeasy Mini Kit (Qiagen, Valencia, CA). From this, aliquots of total miRNA (2 µg) were used for reverse transcription. Analysis of miR-200a, miR-7, miR-96, miR-145, miR-148, miR-195, miR-16, miR-15a, miR-15b, and pre-miR-16 expression were conducted using a 7900HT Fast Real-time PCR system (Applied Biosystems) and a miScript PCR kit (Qiagen). The primers for each miRNA were purchased from Invitrogen, and U6 was used as a control. At the end of the analyses, cycle threshold (Ct) values were determined, and the relative expression of each miRNA was calculated using the values of 2^-ΔΔCT^.

***[^35^S]-Methionine pulse assays***

The indicated cells were incubated for 1 hr at 37°C in methionine-cysteine-free DMEM containing 2% FBS and 10 μM MG132 (all Gibco, Grand Island, NY, USA). After this period, the cells were then incubated in 2% FBS methionine-cysteine-free DMEM containing [^35^S]-methionine/cysteine (250 µCi/sample, Trans [^35^S]-label; ICN) for varying periods. At each time point, cells were extracted on ice with lysis buffer (Cell Signaling Technology, Beverly, MA, USA) containing complete protein inhibitor mixture (Roche, NJ, USA). After determining protein concentration using Nanodrop 2000, an aliquot of the total lysate (≈500 mg protein) was placed into a column containing anti-JNK2 antibody-conjugated agarose beads (R&D Systems, Minneapolis, MN) and incubated overnight at 4°C. All immuno-precipitated samples were then washed with cell lysis buffer for five times, heated at 100°C for 5 min, and then subjected to SDS-PAGE electrophoresis. The presence of [^35^S]-JNK2 was then evaluated (and relative levels quantified) using a PhosphorImager System (Molecular Dynamics, Kent City, MI, USA).

***Cell migration and invasion assay***

Control inserts without matrigel and permeable support for 24-well plates with 8.0 μm transparent PET membranes were purchased from Corning Incorporated (Corning, NY, USA). The invasion kit used here was purchased from BD Biosciences (Bedford, MA, USA). The invasion assay was performed according to manufacturer protocols. In brief, T24T and UMUC3 cells were seeded to chamber inserts in triplicate in 500 μl medium/0.1%FBS. The inserts were then placed into wells containing 1 ml 1:1 mixture of DMEM/Ham’s F12/5%FBS or DMEM/10% FBS. Cells were incubated for 24 hr at 37°C. Thereafter, cells on both the inside and outside of the chamber were fixed with 3.7% formalin for 2 min, washed twice with 1× phosphate-buffered saline (PBS, PH 7.4), treated with 100% methanol for 20 min, washed twice again and then stained with Giemsa (1:20 [v/v] in PBS) for 30 min in the dark. The cells were again washed twice, and then non-invaded cells were scraped away with a water-wetted cotton swab. Images of remaining cells were then taken using an Olympus DP71 system (Olympus America Inc. Center Valley, PA, USA), and the number of the cells in each image was counted using Image J software (NIH, Bethesda, MD, USA). Invasion rate was normalized with the insert control. Data was presented as the percentage of invasion through the BD Matrigel^TM^, matrix and membrane relative to the migration of cells through uncoated membrane. The data shown are representative of three independent experiments.

***Cell Proliferation assay***

T24T (Vector) and T24T (miR-200a) cells were trypsinized, and 1 × 10^3^ of viable cells suspended in 100 μL DMEM/5% FBS were added to each well of 96-well plates. The plates were incubated at 37°C in a humidified atmosphere of 5% CO_2_. Twelve hours later, cells were lysed with 50 μL lysis buffer, and the proliferation of the cells was measured using CellTiter-Glo Luminescent Cell Viability Assay kit (Promega, Madison, WI, USA) with a luminometer (Waltham, MA, USA). The results are expressed as luciferase activity relative to control 12 hrs. (Relative proliferation index).

**References**

1 Qin H, Sun Y, Benveniste EN. The transcription factors Sp1, Sp3, and AP-2 are required for constitutive matrix metalloproteinase-2 gene expression in astroglioma cells. *The Journal of biological chemistry* 1999; 274: 29130-29137.

2 Zhang D, Song L, Li J, Wu K, Huang C. Coordination of JNK1 and JNK2 is critical for GADD45alpha induction and its mediated cell apoptosis in arsenite responses. *The Journal of biological chemistry* 2006; 281: 34113-34123.

3 Zhang HH, Huang B, Cao YH, Li Q, Xu HF. Role of 5-Aza-CdR in mitomycin-C chemosensitivity of T24 bladder cancer cells. *Oncol Lett* 2017; 14: 5652-5656.

4 Huang C, Zeng X, Jiang G, Liao X, Liu C, Li J *et al*. XIAP BIR domain suppresses miR-200a expression and subsequently promotes EGFR protein translation and anchorage-independent growth of bladder cancer cell. *Journal of hematology & oncology* 2017; 10: 6.

5 Liang Y, Zhu J, Huang H, Xiang D, Li Y, Zhang D *et al*. SESN2/sestrin 2 induction-mediated autophagy and inhibitory effect of isorhapontigenin (ISO) on human bladder cancers. *Autophagy* 2016; 12: 1229-1239.

6 Jin H, Xu J, Guo X, Huang H, Li J, Peng M *et al*. XIAP RING domain mediates miR-4295 expression and subsequently inhibiting p63alpha protein translation and promoting transformation of bladder epithelial cells. *Oncotarget* 2016; 7: 56540-56557.

7 Hua X, Xu J, Deng X, Xu J, Li J, Zhu DQ *et al*. New compound ChlA-F induces autophagy-dependent anti-cancer effect via upregulating Sestrin-2 in human bladder cancer. *Cancer Lett* 2018; 436: 38-51.

8 Liu J, Zhang D, Mi X, Xia Q, Yu Y, Zuo Z *et al*. p27 suppresses arsenite-induced Hsp27/Hsp70 expression through inhibiting JNK2/c-Jun- and HSF-1-dependent pathways. *The Journal of biological chemistry* 2010; 285: 26058-26065.

9 Zhu J, Zhang J, Huang H, Li J, Yu Y, Jin H *et al*. Crucial role of c-Jun phosphorylation at Ser63/73 mediated by PHLPP protein degradation in the cheliensisin a inhibition of cell transformation. *Cancer prevention research (Philadelphia, Pa)* 2014; 7: 1270-1281.

10 Hua X, Huang M, Deng X, Xu J, Luo Y, Xie Q *et al*. The inhibitory effect of compound ChlA-F on human bladder cancer cell invasion can be attributed to its blockage of SOX2 protein. *Cell Death Differ* 2019.

11 Xie Q, Guo X, Gu J, Zhang L, Jin H, Huang H *et al*. p85alpha promotes nucleolin transcription and subsequently enhances EGFR mRNA stability and EGF-induced malignant cellular transformation. *Oncotarget* 2016; 7: 16636-16649.

12 Huang H, Jin H, Zhao H, Wang J, Li X, Yan H *et al*. RhoGDIbeta promotes Sp1/MMP-2 expression and bladder cancer invasion through perturbing miR-200c-targeted JNK2 protein translation. *Molecular oncology* 2017; 11: 1579-1594.
